# The Book World of Medicine and Science

**Published:** 1905-05-13

**Authors:** 


### The Book World of Medicine and Science.

Errors of Refraction and their Treatment: A Clinical Pocket-book for Practitioners and Students. By Charles Blair, M.D., F.R.C.S., Surgeon to the Western Ophthalmic Hospital, London, etc. (Bristol: John Wright and Co. 1905. Pp. 102. Price 2s. 6d.

We confess to a wholesome dread of what are sometimes called by their authors small books on great subjects; and Dr. Blair has not furnished us with any reason for altering that general view. He has put into very compressed form a few elementary details concerning refraction, and he must be taken to suggest that this is sufficient for the medical profession, to which his book is apparently addressed. We need only add that the author splits his infinitives, and that he accomplishes the remarkable physical feat of being "under" circumstances, which we had always supposed to be "things that stand around." Also, he says that the smallest image which can be perceived by the most sensitive human retina is found to be one which subtends an angle of about 50″ at the nodal point of the eye, presumably meaning 50‴.

The Food Inspector's Hand-Book. By Francis Vacher, M.O.H. (London: The Sanitary Publishing Company. Pp. 223. Fourth edition. 1905. Illustrations 58. Price 3s. 6d. net.)

The fourth edition of this handbook has just been published, and the size increased by 35 pages, owing to an extra chapter on statutory powers and sundry additions due to revision. The author gives the qualifications of a food-inspector and the chief laws relating to the sale of food, and then describes in detail the physical signs by which unwholesome food, intended for human consumption, may be detected. All foods mentioned in the Public Health Acts are dealt with, also many more not so mentioned. The book thus forms a full and reliable guide for food-inspectors, to whom we can commend it. The illustrations are clear, and add to the value of the work.

How to Keep "Fit," or the Sailor's Guide to Health in all Parts of the World. Compiled by Fleet Surgeon W. G. Barnes, M.D. (London: Gale and Polden. Price 3d.)

We agree with Lord Charles Beresford, who writes of this unpretending booklet: "I should like to see one in the bag of every man on board a man-of-war." We, however, go further, and say that all sailors, naval, mercantile, or in private employment, should possess it. Jack Tar is a practical man, and he will appreciate the direct and concise manner in which the health axioms he is so liable to ignore are explained. There is a commendable absence of technical phraseology, but at the same time a sound, scientific basis at the foundation of the recommendations is apparent. The reason of the advice is simply stated, and, appealing to the intelligence, renders it likely to be followed. A surprising amount of useful information is compressed into the 37 little pages of "How to Keep 'Fit.'"

Income Tax Tables for the Use of Public Companies Bankers, Solicitors, and Others. By G. H. Whybrow, of the Income Tax Repayment Branch, Somerset House. (London: Stevens and Son Limited, Chancery Lane. Price 5s.)

This is a serviceable and practical little work for all who have occasion to make or receive payments subject to the deduction of income tax. It commences with three tables of average rates of income tax for one year, six months, and three months, from 1900 to April 1905, which, with an income tax which has varied during a financial year greatly assists calculation. The rest of the tables are arranged in an ascending scale of farthings from 8d. to 9d. tax on from £1 to £10,000 ascending by £1.

Foods and Dietaries: A Manual of Clinical Dietetics. By R. W. Burnet, M.D., F.R.C.P. Fourth edition. Pp. 200. (Charles Griffin and Co., Ltd. 1905. Price 4s.)

The fact that three editions of this work have been exhausted in a comparatively short time is proof in itself of the merit and utility of Dr. Burnet's book. The author takes each disease separately, and after some general remarks as to foods, goes on to give exact instructions for the feeding of the patient. Suggestions as to the times of meals, the quantity and kind of food at each, along with suitable beverages, are all supplied with admirable skill and judgment. Perhaps rather more space might have been devoted to the dietary in children's diseases, for Dr. Burnet only gives one chapter on this part of the subject, still what is given is clear and to the point. The book closes with an appendix containing 97 recipes for sick-room cookery, and with this collection the doctor and nurse need never be at a loss for a dish to tempt the most capricious appetite. Practitioners will find Dr. Burnet's work invaluable in the treatment of disease and the management of patients and invalids.

Elementary Microscopy. By F. Shillington Scales. (London: Baillière, Tindall and Cox. 1905. Price 3s.)

From a medical point of view this book is very elementary. It deals almost entirely with the microscope itself; only a few pages are given to outline the simpler details of the method of preparing an object for examination. Anyone intending to choose a microscope for himself would do well to read this book. There is an extensive bibliography of books in the English language on the microscope.

City of London Directory, 1905. Published by Messrs. W. H. L. Collingridge. Price 12s. 6d.

We have received a copy of the 1905 edition of this useful work of reference. It contains, as usual, information concerning the City Corporation, Committees, City Officials, and Livery Companies, besides a Streets Guide, a Trades Guide, Alphabetical Directory, and a coloured map of the City of London, showing the latest improvements and alterations. A new and important feature this year is the inclusion of London County Council Committees. In view of the many important questions which these committees have to consider, the names of the members selected to serve on them should prove exceedingly useful, especially so as they are not published officially. The Directory altogether is a most useful work, and a copy should be in the possession of every business man in the City of London.

